# Delving into the Correlation between Magnetic and Lattice Degrees of Freedom from Magnetocaloric and Magnetovolume Effects in Lu_2_Fe_17_ Ribbons

**DOI:** 10.1021/acs.jpcc.5c04207

**Published:** 2025-10-07

**Authors:** J. L. Garrido Álvarez, M. L. Arreguín-Hernández, C. Echevarría-Bonet, Pedro Gorria, I. Puente-Orench, F. Fauth, Jesús A. Blanco, J. L. Sánchez Llamazares, Pablo Álvarez-Alonso

**Affiliations:** † Departamento de Física, 16763 Universidad de Oviedo, 33007 Oviedo, Spain; ‡ Centro de Nanociencias y Nanotecnología, Universidad Nacional Autónoma de México, AP 14, Ensenada 22860, Baja California, Mexico; § IUTA, Universidad de Oviedo, 33203 Gijón, Spain; ∥ Institut Laue-Langevin, 71 Ave des Martyrs, CS 20156, 38042 Grenoble cedex 9, France; ⊥ ALBA Synchrotron Light Source, 08290 Cerdanyola del Vallès, Barcelona, Spain; # 42628 Instituto Potosino de Investigación Científica y Tecnológica A.C., San Luis Potosí S.L.P. 78216, Mexico

## Abstract

Nowadays, R_2_Fe_17_ (R = rare earth) materials with zero (ZTE) or negative (NTE) thermal expansion are of significant interest in advanced applications, especially for intermediate performance low-cost magnets. Lu_2_Fe_17_ ribbon flakes were fabricated by means of the melt-spinning technique, while a bulk sample was synthesized by arc melting and long-term annealing as a reference alloy. Both the as-cast ribbons and the bulk sample adopt a Th_2_Ni_17_-type hexagonal crystal structure. The anomalous temperature dependence of the lattice parameters in the ribbons confirms the existence of strong magnetovolume effects, characterized by NTE and ZTE along the *c* and *a* crystallographic axis, respectively. In addition, magnetic measurements show two magnetic phase transitions, from paramagnetic to helimagnetic and from helimagnetic to a fan structure, with transition temperatures differing between ribbon (*T*_HEL_ = 276 K and *T*_FAN_ = 252 K) and bulk (273 and 257 K) samples, respectively. These differences can be attributed to variations in the exchange interactions caused by slightly different interatomic distances between the Fe atoms. The isothermal entropy change versus temperature curves, Δ*S*_M_(*T*), measured under low magnetic field values (up to 150 mT), provide clear evidence of the existence of a double peak, thus confirming the two successive magnetic phase transitions that occur in Lu_2_Fe_17_ ribbon flakes.

## Graphical abstract

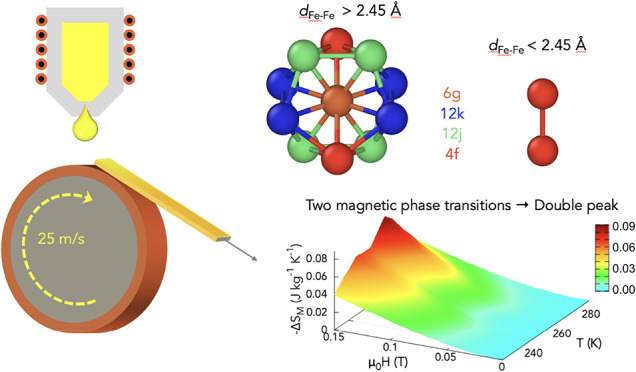

## Introduction

Fe-rich R_2_Fe_17_ (R = rare earth) alloys constitute a family of binary R-T (T = transition metal) intermetallic compounds widely studied in the field of permanent magnets, with notable examples such as Sm_2_Fe_17_N_*x*_.^1–3^ In recent years, the interest in this family has been renewed due to their intriguing magnetic phenomena, which arise from the competition of 3d and 4f magnetism and crystal field interactions, leading to the existence of strong magnetovolume effects.^4–10^ In particular, attention has been paid to studying the magnetocaloric (MC) effect and the nearly-ZTE over a broad temperature interval for several compounds of this series.^10–16^ In the present case, the latter is attributed to magnetovolume effects driven by the dependence of the exchange coupling on the interatomic distances.^17–21^ The MC effect, currently a highly active and cutting-edge research topic,^22–27^ refers to the changes in isothermal magnetic entropy (Δ*S*_M_) and adiabatic temperature (Δ*T*_ad_) induced by a magnetic field. Both temperature variations exhibit a maximum for a given magnetic field around the magnetic ordering temperature of the material.^22^

Depending on the rare earth, R_2_Fe_17_ compounds adopt either a rhombohedral Th_2_Zn_17_-type (space group *R*3̅*m*) or a hexagonal Th_2_Ni_17_-type (*P*6_3_/*mmc*) crystal structure. In these alloys, the R atoms occupy the 6c (2b-2d) and the Fe atoms occupy the 6c-9d-18f-18h (4f-6g-12j-12k) Wyckoff positions in the rhombohedral (hexagonal) cell, respectively.^28^ There are, however, a couple of exceptions: Ho_2_Fe_17_ and Lu_2_Fe_17_, which can crystallize in the disordered Th_2_Ni_17_-type. In this structure, the R atoms occupy the 2b (0, 0, 1/4) and may also be distributed across the 2c (1/3, 2/3, 1/4) and 2d (1/3, 2/3, 3/4) crystallographic sites, while the Fe atoms occupy 4f (1/3, 2/3, *z*), 6g (1/2, 0, 0), 12j (*x*, *y*, 1/4), and 12k (*x*, 2*x*, *z*) sites.^18,29^ In the specific case of Lu_2_Fe_17_, the 2b site is partially occupied by Lu atoms, and additional pairs of Fe atoms commonly appear in positions 4e (0, 0, *z*),^30^ leading to a shift in equilibrium composition from Lu_2_Fe_17_ to Lu_2_Fe_19_ at ambient pressure.^31^

The magnetic phase diagram of this family of alloys is remarkably rich, with most magnetic phenomena strongly influenced by the interatomic distances between Fe atoms, *d*_Fe–Fe_, especially those occupying the dumbbell sites (6c or 4f). These sites show short bond lengths, often below the threshold for parallel coupling of the Fe spins (*d*_Fe–Fe_ ≈ 2.45 Å).^32^ Investigating the effects of the Fe–Fe distances on the magnetic properties of Lu_2_Fe_17_ is particularly interesting due to the nonmagnetic nature of the Lu atom, which enables the isolation of intrinsic magnetic interactions within the Fe network. Furthermore, Lu_2_Fe_17_ stands out among the R_2_Fe_17_ alloys as the compound with the smallest unit cell volume, attributed to the very short Fe–Fe distances. This structural feature also leads to a comparatively low ordering temperature relative to other R_2_Fe_17_ compounds. In addition, Lu_2_Fe_17_ exhibits an intermediate saturation magnetization, i.e., lower than that of the ferromagnetic members of the series but higher than that of the ferrimagnetic ones. Neutron diffraction investigations provide a unique picture of the magnetic phase diagram of this alloy.^33^ A dominant ferromagnetic (FM) interaction occurs within the layers oriented perpendicular to the *c*-axis of the hexagonal unit cell, coexisting with antiferromagnetic (AFM) components along the *c*-axis. This interplay results in a fan-type magnetic arrangement, where the angle between consecutive layers varies with temperature. Several transitions from a low-temperature FM to an incommensurate AFM or fan, and then to a helical magnetic structure have been reported.^33–35^ Moreover, the ferromagnetic ground state of Lu_2_Fe_17_ was shown to be suppressed under hydrostatic pressure and uniaxial compression along the *c*-axis.^30,31,33,34^ The origin of this complex magnetic phase diagram lies in the strong dependence of exchange interactions on the Fe–Fe interatomic distance (*d*_Fe–Fe_). When *d*_Fe–Fe_ < 2.45 Å, the Fe spins couple antiparallel, consistent with the Bethe–Slater curve, which also explains the nearly zero (ZTE) or even negative (NTE) thermal expansion observed below the Néel temperature.^32–35^

The conventional fabrication processes (by means of arc or induction melting techniques) of these alloys require prolonged (at least several days) heat treatments at high temperatures (usually above 1200 K) to guarantee the formation of the 2:17 crystal structure. In contrast, rapid solidification from the melt (as fast as 10^6^ K/s) provides an extraordinarily efficient method to fabricate single-phase R_2_Fe_17_ alloys in a single-step process, avoiding long-time high-temperature annealing, and providing a cost-effective material production. Additionally, this method ensures a highly homogeneous material in terms of its chemical composition.^15,36,37^ In this work, we report on the crystal structure and microstructure of new Lu_2_Fe_17_ ribbons synthesized by rapid solidification and their magnetic properties, paying special attention to the temperature dependence of magnetovolume (ZTE and NTE) and magnetocaloric effects. We compare the current results with those obtained for a bulk alloy.

## Experimental Methods

### Sample Preparation

Two bulk stoichiometric Lu_2_Fe_17_ alloys were synthesized by arc melting from pure elements (Fe, 99.97%, and Lu, 99.9%, supplied by Alfa Aesar) in a MAM-1 mini arc melter from Edmund Bühler GmbH. The samples were remelted several times to ensure their starting chemical homogeneity. One ingot was thermally annealed for 1 week at 1173 K to achieve a highly ordered homogeneous Th_2_Ni_17_-type crystal structure, while the other one was used as a precursor for producing ribbon flakes via induction melting under an ultrahigh-purity Ar atmosphere in a model SC melt spinner system (Edmund Bühler GmbH) at a copper wheel linear speed of 20 m/s.

### Sample Characterization

The microstructure and elemental chemical composition of melt-spun ribbons were analyzed using a FEI SEM-QUANTA-250 scanning electron microscope (SEM) equipped with an energy-dispersive X-ray spectroscopy (EDS) system. Through room-temperature X-ray powder diffraction (XRD), we determined the crystalline structure. Laboratory XRD patterns were collected in a PANalytical X’Pert PRO diffractometer using a Cu anode (λ = 1.5406 Å) in the 2θ range 10°–140°. High-resolution XRD patterns were collected from finely powdered samples using synchrotron radiation (λ = 0.4428 Å) over a 2θ range 5°–25°, with a 2θ step increment of 0.1°, on the diffractometer BL04-MSPD at ALBA synchrotron (Spain). The diffraction patterns were analyzed using the FullProf suite package based on the Rietveld method.^38,39^ Neutron diffraction patterns were recorded using the XtremeD powder diffractometer at the Institute Laue-Langevin (France) in the temperature range of 12–480 K. Magnetization measurements were performed using vibrating sample magnetometry in a Dynacool PPMS system from Quantum Design. Magnetization *M*(*T*) curves were recorded at a temperature sweep rate of 1.0 K/min, under applied magnetic fields up to 5 T.

## Results and Discussion

### Microstructure and Composition of the Ribbons

Typical SEM images of the cross-section and the ribbon surfaces, those in contact with the copper wheel (CS) and those not in contact (NCS), for the Lu_2_Fe_17_ ribbons are shown in Figure 1. The mean ribbon thickness, estimated from the cross-sectional SEM image, was 38(4) μm. A significant difference in the microstructure of the ribbon surfaces was observed: while the grain size on the CS side is in the nanoscale range, grains on the NCS side grew to sizes on the order of microns. Higher-magnification imaging revealed that grains on the CS side can be as small as 200 nm. The fast solidification process induced by the high cooling rate, typically on the order of 10^6^ K/s, leads to this particular microstructure. Figure 2(a) displays a representative EDS spectrum of Lu_2_Fe_17_ melt-spun ribbons. Multiple EDS spectra were collected in various regions of the ribbon samples, all confirming a composition close to 1.7:17 stoichiometry (Lu 9% and Fe 91%). Figure 2(b) shows the area of the sample where EDS element mapping images for Lu and Fe were collected. Both Lu and Fe are homogeneously distributed throughout the sample, as illustrated in the mapping images.

**Figure 1:**
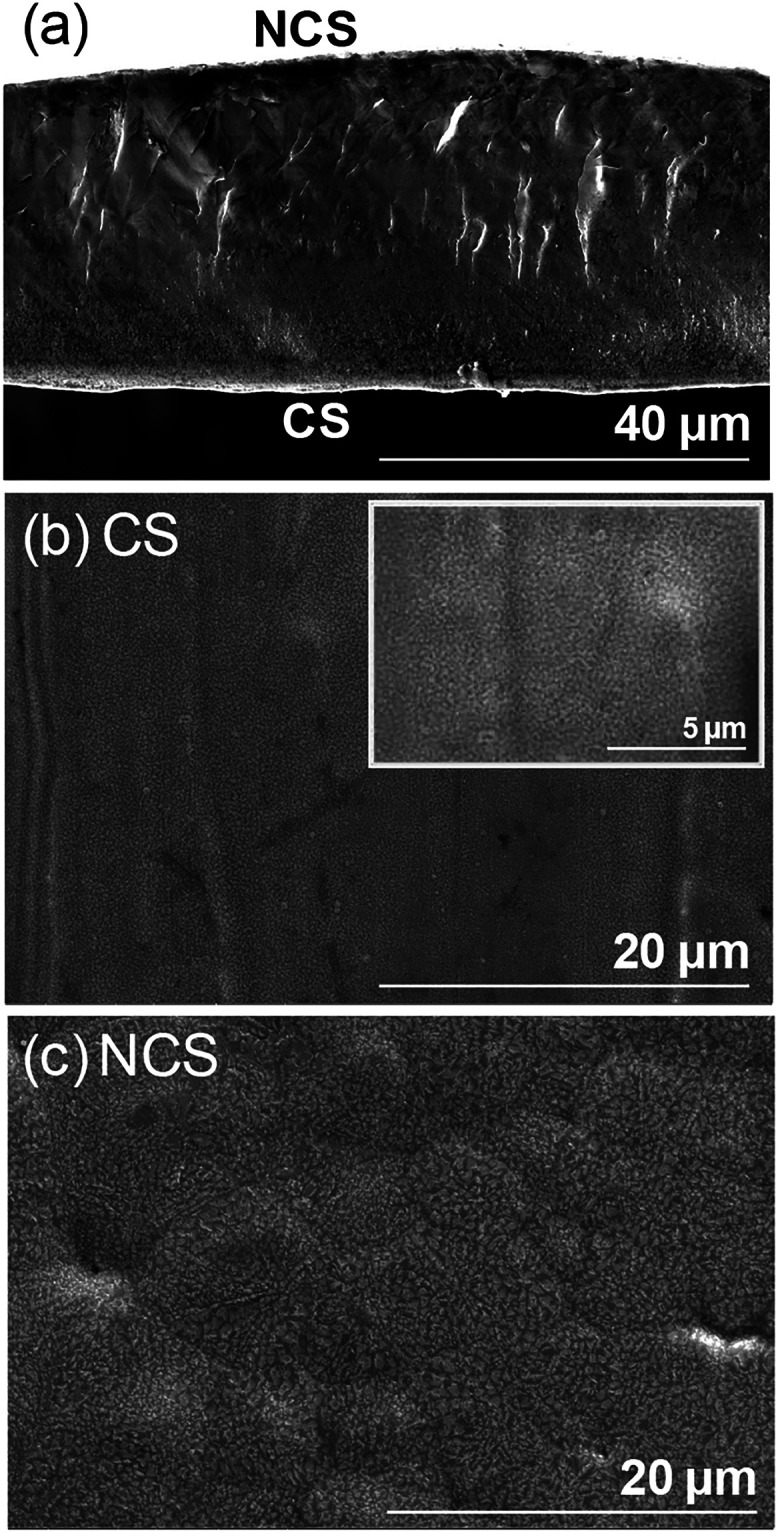
SEM micrographs of (a) ribbon cross-section, (b) contact surface, and (c) the noncontact surface. The image inserted in (b) was taken at a higher magnification to show the granular microstructure at the noncontact surface.

**Figure 2:**
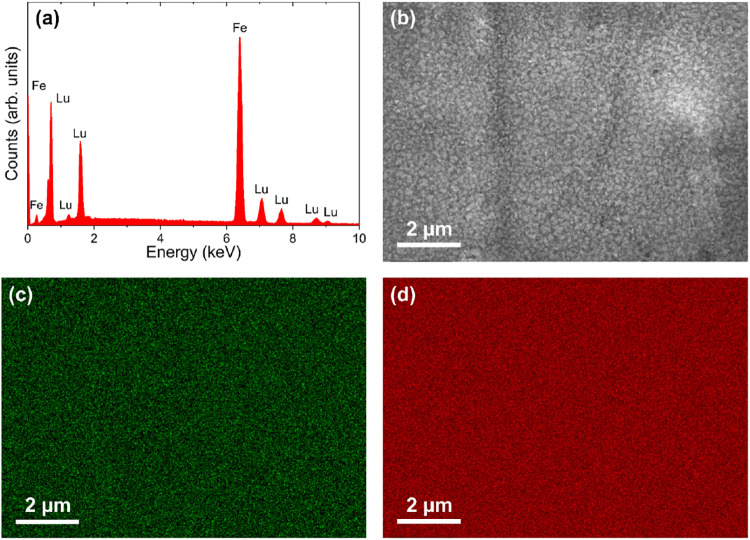
(a) EDS spectrum of Lu_2_Fe_17_ melt-spun ribbons. (b) SEM micrograph of the area where EDS element mapping images for (c) Lu and (d) Fe were taken.

### Crystal Structure

Figure 3 shows the experimental and calculated XRD patterns for ribbon and bulk samples and the high-resolution synchrotron pattern for the Lu_2_Fe_17_ ribbons. To start with, it is important to remark that the ribbon sample is a single-phase alloy, whereas the bulk one contains a small amount of α-Fe impurity (<2 wt %). In both samples, the Lu–Fe phase adopts the hexagonal Th_2_Ni_17_-type crystal structure. These results confirm that the formation of a polycrystalline single-phase alloy with a hexagonal crystal structure is straightforward by using the rapid quenching technique. A comparison of the XRD patterns reveals a noticeable broadening of the diffraction peaks for the patterns corresponding to the ribbons. The particular microstructure generated in the ribbons during the rapid-solidification process leads to this broadening, as already observed in R_2_Fe_17_ melt-spun ribbons with R = Pr and Nd synthesized under similar conditions.^15,36^

**Figure 3:**
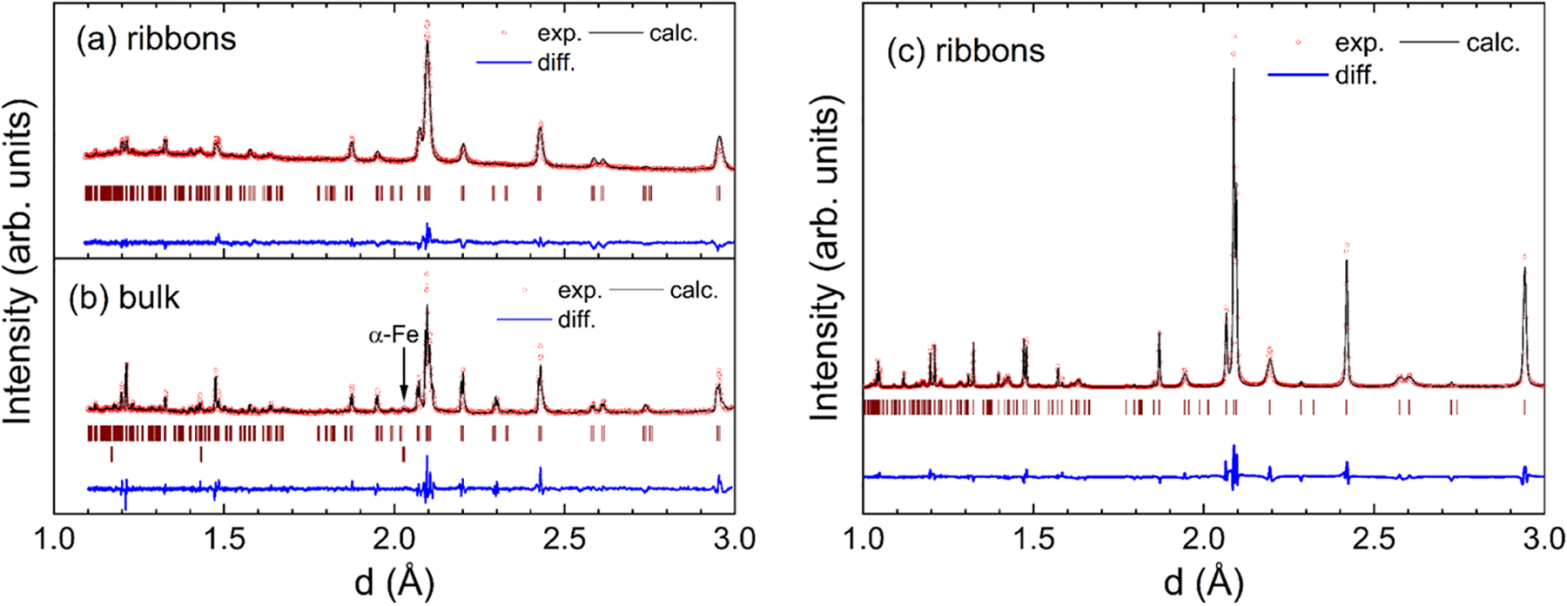
Laboratory XRD patterns for (a) melt-spun ribbons and (b) bulk alloy. (c) High-resolution synchrotron XRD pattern for the melt-spun ribbons. The experimental data and the calculated pattern are depicted as red points and a continuous black line, respectively. The blue line is the difference between the experimental and calculated patterns, and the Bragg reflections are indicated by vertical lines.

To properly fit the diffraction patterns, we have initially assumed the standard four Wyckoff positions for Fe atoms in the hexagonal crystal structure. However, the fit does not account adequately for the intensity of several Bragg peaks. Thus, it was necessary to consider other Wyckoff positions for Fe, 4e, and the 2b shared with Lu (also located on 2d) of the *P*6_3_/*mmc* crystal structure.^28^ Although the fit was more satisfactory in the latter case, it yielded unphysical interatomic distances, being lower than 2–2.2 Å. Hence, other attempts were undertaken, like the one proposed by Givord et al.^31^ but, again, too small distances among Fe–Fe atoms were obtained. Therefore, in this case, the ordered hexagonal Th_2_Ni_17_-type structure was taken as the optimal solution. Table 1 summarizes and compares the crystallographic information on the Lu_2_Fe_17_ melt-spun ribbons with that of the bulk sample (i.e., lattice parameters, atomic positions, and site occupations).

**Table 1:** Crystallographic Information for the Bulk Sample and Melt-Spun Ribbons in the Hexagonal Cell Choice (See Text for More Details)

			ribbons	bulk
Lu_2_Fe_**17**_ Space group: *P*6_3_/*mmc*	a (Å)		8.4050(2)	8.4009(1)
	c (Å)		8.2921(2)	8.2736(1)
	Fe 4f	Z	0.6085	0.6049
		Occ (%)	100	100
	Fe6g	Occ (%)	83.8	82.6
	Fe12j	X	0.3518	0.3573
		Y	0.0135	0.0334
		Occ (%)	100	100
	Fe12k	X	0.1657	0.1662
		Z	0.0145	0.0235
		Occ (%)	100	100
	Lu2b	Occ (%)	75.1	72.3
	Lu2c	Occ (%)	100	100
α-Fe Space group: *Im*3̅*m*	a (Å)		-	2.8641(3)
R_B_ (%)			12.0	15.8
χ^2^			2.80	6.28

Table 2 lists the first, second, and third neighbors’ interatomic distances at the Wyckoff positions. The values for the cell parameter are similar to those found for the reference bulk alloy in this work and agree with previously reported values.^30,31^ However, a significant deficiency of Lu was observed at the 2b crystallographic site,^29^ which provokes the migration of Fe atoms from the 4f to the 2b crystallographic sites.^41^ Therefore, the Lu content is reduced compared with the nominal composition, implying that the actual composition is close to Lu_1.7_Fe_17_ (consistent with EDS results). Additionally, a shortening of the Fe 4f-Fe 4f (dumbbell sites) distances is found for the ribbons (see Table 2), being below 2.45 Å, which will increase the dumbbell interaction and cause the magnetic moments of the Fe atoms to couple antiparallel.^34^ The distances between adjacent atomic layers are below 2.45 Å for 4f-4f and 6g-12k atom pairs and above 2.45 Å for 12k-12j and 6g-12j (see Table 2), implying competitive positive and negative magnetic interactions, respectively. The latter produces spin frustration in adjacent atomic layers, inducing different magnetic phenomena.

**Table 2:** Selected Interatomic Distances (Å) in Bulk and Ribbon Samples

Wyckoff pairs	ribbon	bulk
Fe 4f-Fe 4f	2.3475	2.4018
Fe 4f-Fe6g	2.5876	2.5756
Fe 4f-Fe12k	2.6444	2.6543
Fe6g-Fe12k	2.4364	2.4363
Fe6g-Fe12j	2.4506	2.4763
Fe6g-Lu2c	3.1913	3.1874
Fe12j-Fe12k	2.4833	2.5769
Fe12j-Fe12k	2.5882	2.4920
Fe12j-Fe12j	2.6064	2.4396
Fe12k-Fe12k	2.4251	2.4487
Fe12k-Fe12j	2.4838	
Fe12k-Fe12j	2.5877	
Fe12k-Lu2b		3.0589
Fe12k-Lu2c		3.0708
Lu2b-Fe12j	2.9010	2.8712
Lu2b-Fe12k	3.1036	3.3114
Lu2b-Fe12k	3.2614	
Lu2b-Lu2b		4.1368
Lu2c-Fe12j	2.8411	2.9857
Lu2c-Fe 4f	2.9723	2.9359
Lu2c-Fe12k	3.1244	4.7708

### Magnetovolume Effects

Given that Fe–Fe interatomic distances below 2.5 Å indicate negative exchange interactions capable of provoking magnetovolume anomalies, neutron diffraction patterns were collected between 2 and 500 K in both bulk and ribbon samples. Figure 4(a,b) display the profile matching of the neutron diffraction pattern of Lu_2_Fe_17_ ribbons at 12 and 480 K, respectively. In the paramagnetic region, only Bragg diffraction peaks corresponding to the nuclear scattering are observed. However, in the magnetic region, peaks associated with both the nuclear and magnetic coherent scattering are present. In this case, the nuclear peaks overlap with the magnetic ones, which accounts for the difference in intensities observed between the diffraction patterns shown in the inset of Figure 4. Additionally, a slight shift in the diffraction peaks is noticeable, indicating a slight variation in the lattice parameters across the temperature range between the temperature extremes, a hint of the existence of magnetovolume effects.

**Figure 4:**
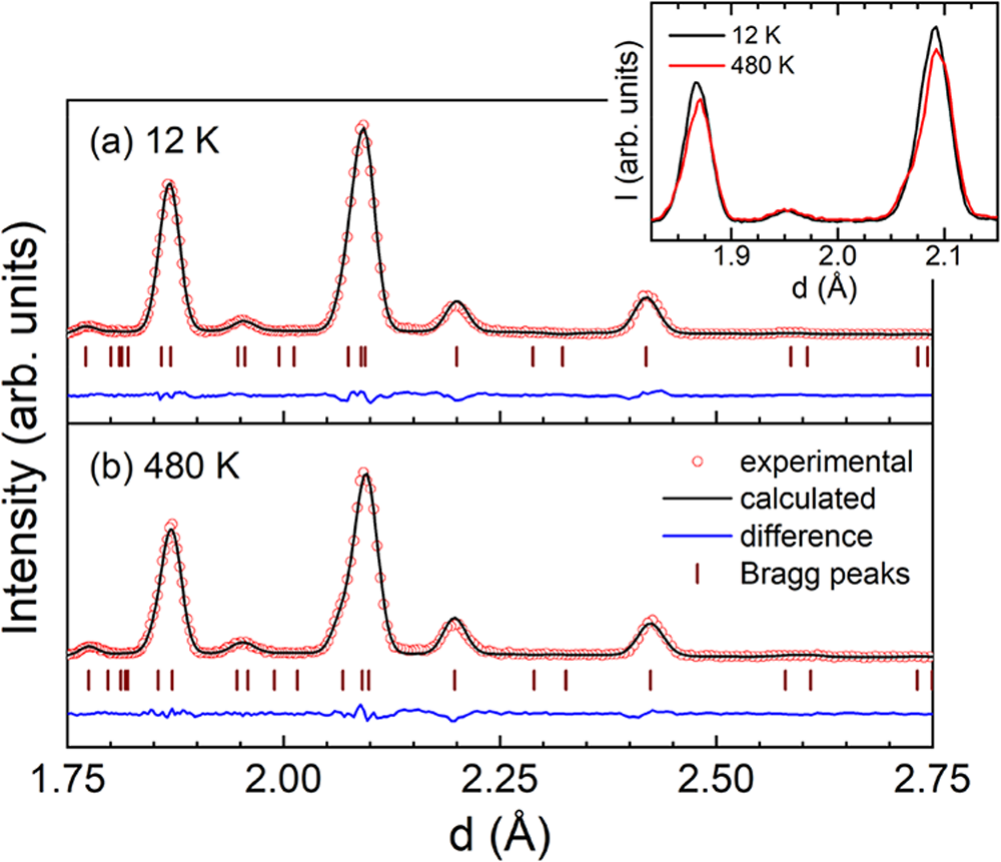
Le Bail fitting of the neutron diffraction patterns for the Lu_2_Fe_17_ melt-spun ribbons at (a) 12 K and (b) 480 K. The inset compares the intensities of the Bragg peaks.

Figure 5(a,b) show the temperature dependences of the cell parameters *a*(*T*) and *c*(*T*) for both Lu_2_Fe_17_ samples across the temperature range of 12 to 480 K, derived from Le Bail analysis of neutron diffraction patterns. Upon heating from *T* = 2 K, three different regions emerge based on the temperature dependence of the *a* and *c* unit cell parameters for the ribbons: (i) nearly constant thermal dependence along the *c*-axis (coefficient of thermal expansion, CTE, α*_c_* = −1.8 × 10^–6^ K^–1^), together with a decrease in the basal-plane unit cell parameters (∼0.12%) up to around *T*_t_ ∼ 80 K, which has been associated with a transition from a high-temperature incommensurate antiferromagnetic phase to a low-temperature ferromagnetic phase when there is deficiency of Lu;^41^ (ii) Invar-like behavior of the unit cell along the *a*-axis (α*_c_* = −1.5 × 10^–6^ K^–1^ in the range 100–280 K) and a strong NTE for parameter *c* (α*_c_* = −13.6 × 10^–6^ K^–1^ in the range 100–220 K).

**Figure 5:**
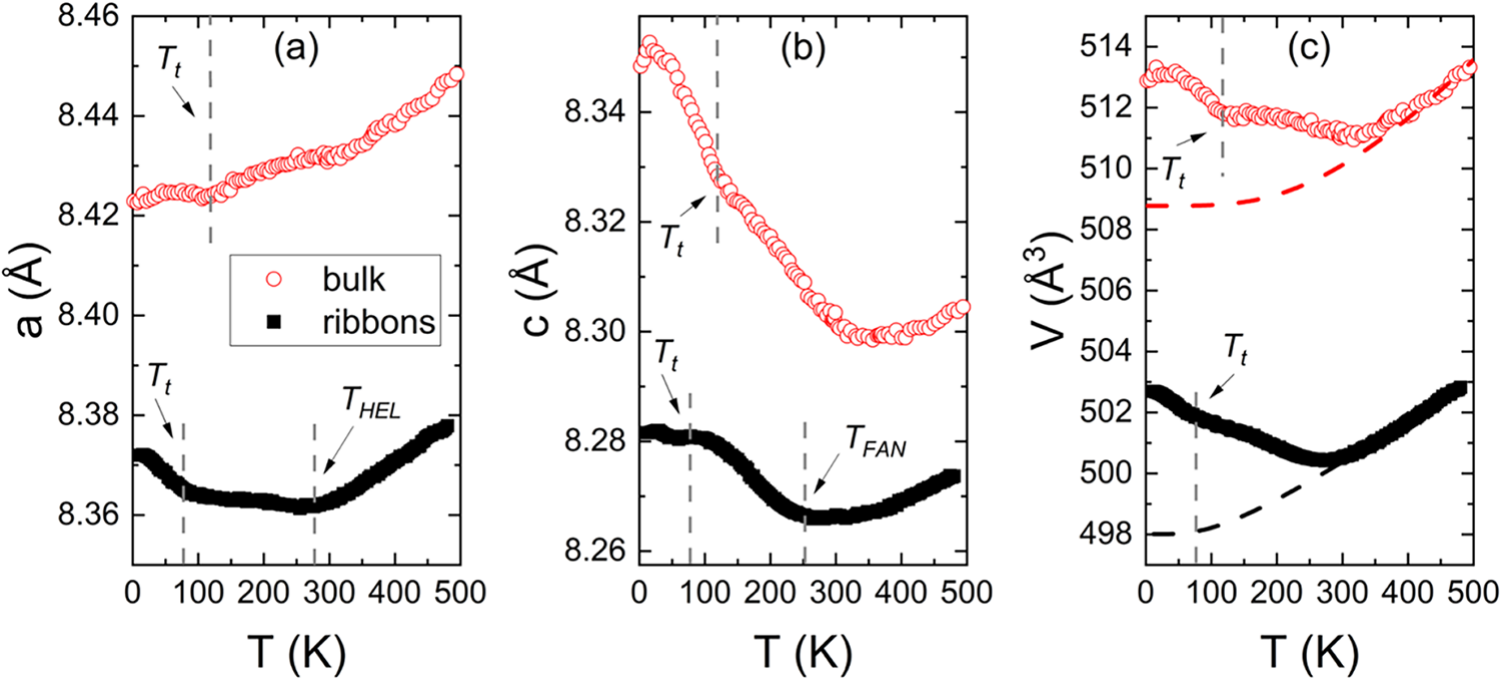
Temperature dependence of unit cell lattice parameters, *a* (a), *c* (b) and the volume *V* (c) for ribbon (black squares) and bulk (red dots) Lu_2_Fe_17_ samples. The vertical gray dashed lines indicate changes in the slope of the curves, which correspond to magnetic phase transitions *T*_HEL_, *T*_FAN_, and *T*_t_ (see text for details). The black and red dashed lines in (c) correspond to the Grüneisen curves for ribbons and bulk alloys, respectively.

The variations in the trends correspond to the two observed magnetic transitions, *T*_HEL_ and *T*_FAN_, respectively (see below); (iii) both *a* and *c* parameters show a Grüneisen-like behavior for temperatures greater than 300 K, approximately. In the case of the bulk specimen, the lattice parameter *a* exhibits nearly zero thermal expansion at low temperatures and then expands, while the lattice parameter *c* contracts until approximately the magnetic ordering temperature. Nevertheless, both the *a* and *c* curves show a slight change in slope at *T*_t_ ∼ 120 K, greater than that found in ribbons. The differences in lattice parameters between the bulk and ribbons and their temperature dependence can be attributed to the slight variations in the Fe–Fe interatomic distances,^41^ as estimated from XRD analysis.

Figure 5(c) compares the temperature dependence of the unit cell volume *V*(*T*) with the simulated phonon-mediated lattice contribution, Grüneisen curve,^42^ which predicts *V*(*T*) if the material is paramagnetic across the entire temperature range. The dependence shows a significant decrease below 275 K, followed by a Grüneisen-like increase, which is typical of metallic compounds above this temperature. At low temperatures, the cell volume is larger than that expected for a nonmagnetic system because the increased distances stabilize the ferromagnetic coupling between the Fe magnetic moments. This indicates that, upon cooling, a volume expansion occurs in association with the onset of magnetic ordering. This coupling between magnetic and lattice degrees of freedom may even be exploited in magnetic refrigeration applications. Significant spontaneous magnetostrictive or magnetovolume deformations characterize R_2_Fe_17_ alloys, and the Fe sublattice primarily drives these effects.^43^ In fact, when heating from a low temperature, a contraction in volume occurs, and the Fe magnetic moments decrease. This weakens the magnetovolume coupling, resulting in volume expansion above the magnetic ordering temperature. The cell volume of the bulk exhibits a similar trend to that observed in the case of ribbons, but a minor deviation is also visible near *T*_t_.

### Magnetic Behavior

Figure 6(a) shows the magnetization *M*(*T*) curves of Lu_2_Fe_17_ ribbons measured during cooling from 350 K under low values of the applied magnetic field (5, 20, 60, and 100 mT). At lower applied field values (20 and 5 mT), a slight shoulder is observed in the thermomagnetic curve. A careful analysis of the temperature derivative of magnetization d*M*/d*T* [Figure 6(c)] reveals the existence of two transitions. These transitions have been previously reported as (i) a transition from the paramagnetic to the helimagnetic state and (ii) a subsequent transition at a lower temperature from the helimagnetic state to a fan magnetic structure, both occurring upon cooling.^44^ In the case of the bulk alloy [see Figure 6(b)], the existence of both transitions is more evident, although the corresponding critical temperatures differ. The critical temperatures associated with Lu_2_Fe_17_ ribbons show that the magnetic phase transitions to a helimagnetic state occur at *T*_HEL_ = 276 K and to a fan structure at *T*_FAN_ = 252 K. In comparison, these transition temperatures for the parental bulk alloy are *T*_HEL_ = 273 K and *T*_FAN_ = 257 K. These critical temperatures were estimated from the minimum of d*M*/d*T*(*T*) at 5 mT, as illustrated in Figure 6(c,d). Both transition temperatures respond strongly to even slight variations in interatomic distances,^45^ where the *T*_FAN_ transition moves to lower temperatures with increasing pressure, disappearing for *P* > 0.5 GPa. Therefore, the difference in transition temperatures can be attributed to variations in the Fe–Fe magnetic interactions caused by differences in interatomic distances between the Lu_2_Fe_17_ bulk and ribbons. As Table 2 indicates, Fe atoms in the dumbbell sites are closer in the ribbons than in the bulk, which results in a stronger AFM character, thus favoring the para-to-helimagnetic transition. At higher applied magnetic field values, the Fe magnetic moments tend to align with the applied magnetic field, leading to a single broad transition.

**Figure 6:**
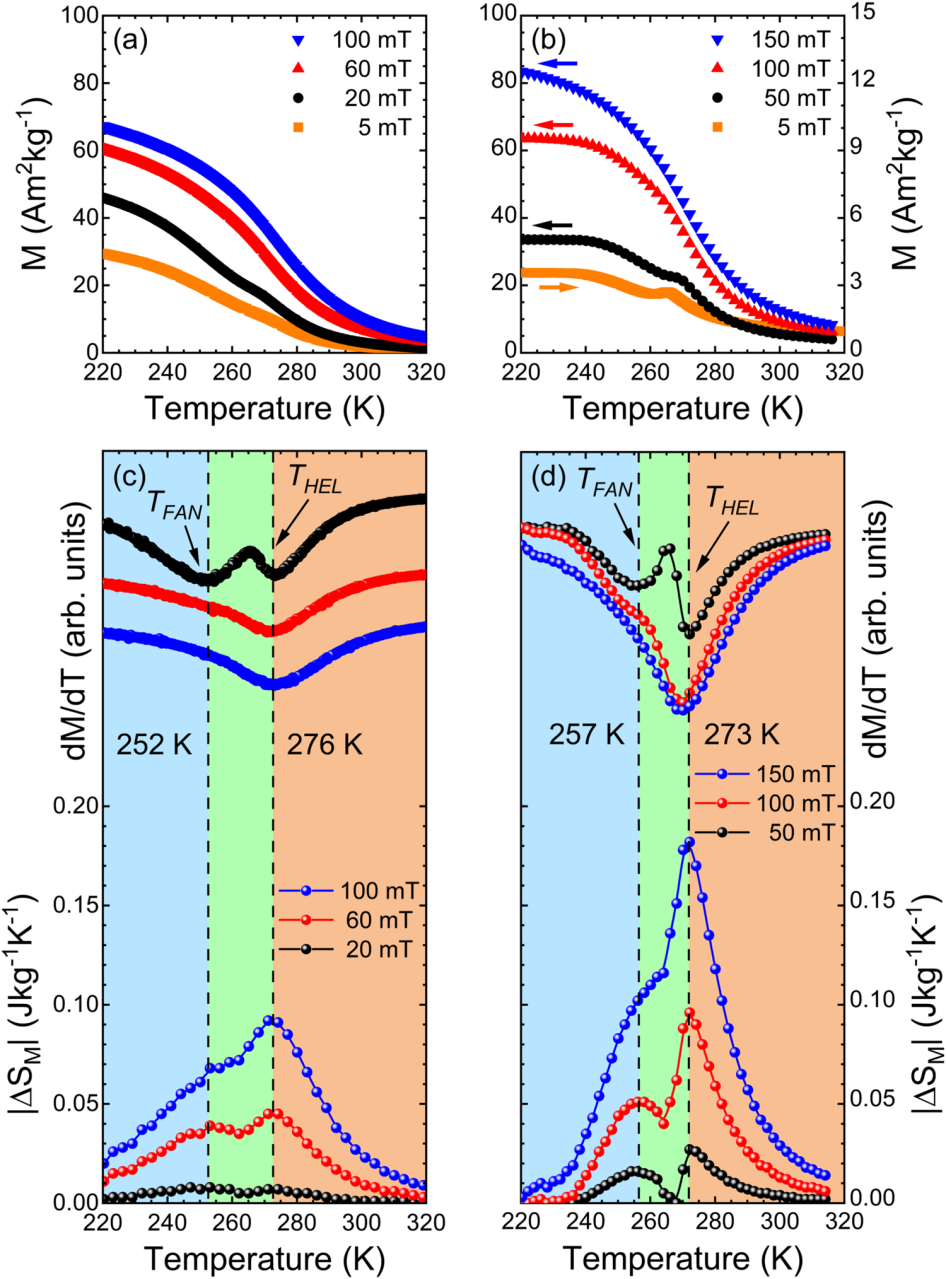
*M*(*T*) curves measured under low magnetic fields for (a) ribbon and (b) bulk Lu_2_Fe_17_ samples. Temperature dependence of d*M/*d*T* [upper part of (c, d)] and |Δ*S*_M_(*T*)| [bottom part of (c, d)] for ribbon and bulk Lu_2_Fe_17_ samples, respectively. Colored rectangles highlight the regions associated with the paramagnetic, helimagnetic, and fan phases. Lines are guides for the eyes.

It is worth noting that the ribbons do not reach the saturation-magnetic state in the temperature range measured; the negative exchange interaction between Fe atoms separated by distances below 2.45 Å can be the reason for this behavior.^35^ Another contributing factor is the existence of the magnetovolume effects characteristic of the R_2_Fe_17_ alloys.^46^ Figure S1 of the Supporting Information file shows the *M*(μ_0_ *H*) curve for both samples at 2 K up to 5 T. We estimated a saturation magnetization *M*_S_ of 131(1) Am^2^kg^–1^ for melt-spun ribbon by fitting the high-magnetic-field region to an approach-to-saturation law.^47^ This value is comparable to that found in the literature^28^ and for the parental bulk alloy, *M*_S_ = 138(1) Am^2^kg^–1^, but smaller than the values reported for ferromagnetic R_2_Fe_17_ (R = Pr, Nd).^12,36,40^ This difference arises because Lu, being a nonmagnetic element, does not contribute to the total magnetic moment.

At temperatures well above *T*_HEL_, the isothermal magnetization curves exhibit the linear behavior characteristic of a paramagnetic state [see Figure S2­(a,c)]. However, it is not perfectly linear at temperatures close to *T*_HEL_, indicating the existence of short-range magnetic order. From the sets of isothermal magnetization *M*(μ_0_ *H*) curves, we also obtained Arrott’s plots [see Figures S2­(b,d)]. The absence of the characteristic S-shape plots, which are indicative of a first-order phase transition, proves the second-order nature of the magnetic phase transition, according to the Banerjee criterion.^48^

### Magnetic Transitions Explored from the Magnetocaloric Effect

Since the magnetic phase transitions are also reflected in the isothermal magnetic entropy change, Δ*S*_M_(*T*), as a function of temperature under low magnetic fields, we used these curves as a complementary tool to characterize and gain insight into the successive magnetic phase transitions experienced by the compound.^49^ Additionally, we obtained the Δ*S*_M_(*T*) curves for magnetic field changes of up to 2 and 5 T. Utilizing the Maxwell relation from the set of isothermal magnetization curves, *M*(μ_0_ *H*), shown in Figure S1­(a,c) of the *Suppl. Info*, we estimate these curves. In particular, the two successive magnetic transitions observed in d*M*/d*T*(*T*) curves [see Figure 6(c,d), upper part] are responsible for the two peaks in the |Δ*S*_M_(*T*)| curves at low values of μ_0_Δ*H* [see Figure 6(c,d), lower part]. The two magnetic transitions are more clearly observed in the low-field isothermal magnetic entropy change than in the temperature derivative of the magnetization, as the double peak disappears in d*M*/d*T*(*T*) curves under low magnetic fields, while it remains visible at higher fields in the Δ*S*_M_(*T*) curves. These two minima in the |Δ*S*_M_(*T*)| curves for Lu_2_Fe_17_ ribbons disappear at lower magnetic fields compared to the bulk material, indicating that the critical field for the transition from fan and helical magnetic structures to the ferromagnetic state is lower in the ribbons than in the bulk alloy. The present findings clearly illustrate the high sensitivity of |Δ*S*_M_(*T*)| to magnetic phase transitions under low magnetic fields.

Figure 7 compares the |Δ*S*_M_(*T*)| curves for bulk and ribbon samples under magnetic field changes μ_0_Δ*H* = 2 and 5 T. These curves exhibit the expected shape for MC materials undergoing a second-order magnetic phase transition, i.e., a broad peak with the maximum, |Δ*S*_M_|^max^, occurring at temperatures close to the critical transition temperatures. It is important to note that the temperature at which |Δ*S*_M_|^max^ occurs differs for bulk and ribbons. Specifically, |Δ*S*_M_|^max^ takes place around the corresponding *T*_HEL_, i.e., at approximately 270 and 276 K for the bulk and the ribbon samples, respectively. Another important observation is the magnetic field dependence of the |Δ*S*_M_|^max^ measured near the ordering point (see inset of Figure 7). Landau’s theory for second-order magnetic phase transitions can describe the |ΔS_M_|^max^ behavior within the mean-field approximation as^49,50^

1 $${\left|{\Delta S}_{M}\right|}^{\max}={A\left(H+H_{0}\right)}^{2/3}-A{H_{0}}^{2/3}+BH^{4/3}$$

where *A* and *B* are due to intrinsic parameters related to the Lu_2_Fe_17_ material, while *H*_0_ is an extrinsic parameter primarily determined by the purity and microstructure of the samples. Although the maximum entropy change grows indefinitely in a strong magnetic field with a dependence on *H* ^2/3^, this is not physically feasible.

**Figure 7:**
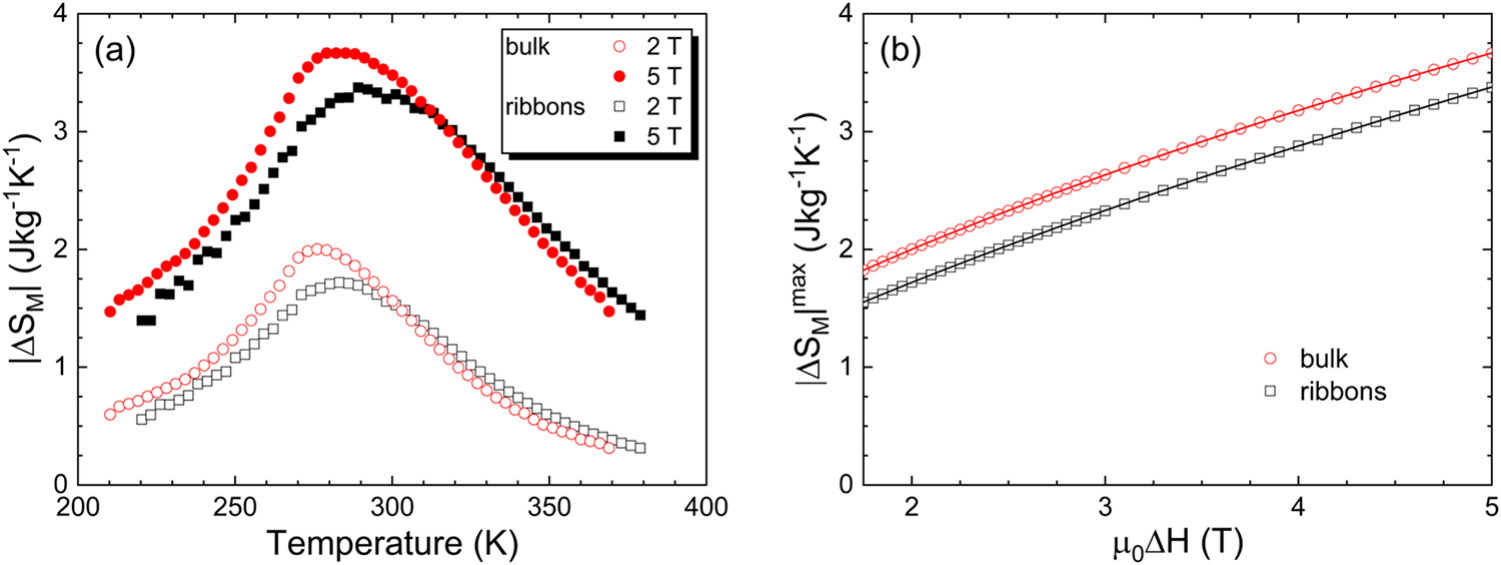
(a) Isothermal magnetic entropy change |Δ*S*_M_| as a function of temperature for bulk and ribbon Lu_2_Fe_17_ samples. (b) Maximum value of the magnetic entropy change, |Δ*S*_M_|^max^, as a function of μ_0_Δ*H*, along with the corresponding fits based on Lyubina’s model (see text for further details).

Therefore, the growth of |Δ*S*_M_|^max^ should slow down as an indication of approaching saturation. Full saturation in the helimagnetic case is unattainable within Landau’s theory, but a trend to saturation can be accounted for by including the two distinct terms of eq 1. In our case, the values obtained from the refinement of the experimental data to eq 1 are *A* = 1.476(7) and 1.337(12) J/(kg·K·T^2/3^), *B* = −0.048(1) and −0.014(1) J/(kg·K·T^4/3^), and *H*_0_ = 0.086(4) and 0.266(14) T for the bulk and ribbon samples, respectively. While the *A* and *B* parameters differ slightly between these two samples, they are overall quite similar. In contrast, the *H*_0_ parameter for the ribbon sample, which is proportional to the distribution of transition temperatures,^49,50^ is significantly larger, indicating a broad distribution of ordering temperatures and strain effects in the ribbon sample, leading to a broader Δ*S*_M_(*T*) curve. This is further corroborated by the increase in the full width at half-maximum (δ*T*_FWHM_ = *T*_hot_ – *T*_cold_) (see Table 3). The values obtained from the fit align with those reported in the literature concerning other magnetic systems undergoing second-order magnetic phase transitions, such as the 2:17 alloy Pr_1.64_Sm_0.36_Fe_17_ or other families of compounds, including Mn_5_Ge_3‑x_Zn_*x*_, Gd_1–*x*_Sm_*x*_Co_2_, and Ni-doped MnCoGe.^51–54^

**Table 3:** Maximum Absolute Value of the Isothermal Magnetic Entropy Change |Δ*S*_M_|^max^, Refrigerant Capacities *RC*-1, *RC*-2, and *RC*-3, *TEC*, and the Temperatures Used to Calculate Them, *T*_hot_, *T*_cold_

	ribbon		bulk	
	2 T	5 T	2 T	5 T
|Δ*S*_M_|^max^ (Jkg^–1^K^–1^)	1.7	3.4	2.0	3.7
*RC*-1 (J/kg)	164	463	163	475
*RC*-2 (J/kg)	127	361	126	367
δ*T*_FWHM_ (K)	95	137	81	130
*T*_hot_ (K)	333	368	321	357
*T*_cold_ (K)	238	231	240	227
*RC*-3 (J/kg)	82	235	83	242
*T*_hot_^*^ (K)	334	370	334	367
*T*_cold_^*^ (K)	238	226	238	214
*TEC*(3 K) (Jkg^–1^K^–1^)	1.7	3.4	2.2	4.1
*TEC*(10 K) (Jkg^–1^K^–1^)	1.6	3.2	2.1	4.0

In Table 3, we present a summary of the most significant MC properties of Lu_2_Fe_17_ bulk and ribbon samples, including the refrigerant capacity (*RC*) and Temperature Averaged Entropy Change (*TEC*), figure of merits for comparing the efficiency of materials in converting the work done by the magnetic field into cooling power. *RC* can be estimated from the Δ*S*_M_(*T*, μ_0_Δ*H*) curves following different approaches (referred to as *RC*-1, *RC*-2, and *RC*-3)^55^

2 $$\mathrm{RC}-1\left(\mu_{0}\Delta H\right)={\left|\Delta S_{M}\right|}^{\max}\times \left(T_{\mathrm{hot}}-T_{\mathrm{cold}}\right)$$

3 $$\mathrm{RC}-2\left(\mu_{0}\Delta H\right)=\int_{T_{\mathrm{cold}}}^{T_{\mathrm{hot}}}\Delta S_{M}\left(T,\mu_{0}\Delta H\right)dT$$

4 $$\mathrm{RC}-3\left(\mu_{0}\Delta H\right)=\max\left\{\Delta S_{M}\left(T_{\mathrm{cold}}^{*},\mu_{0}\Delta H\right)\times \left(T_{\mathrm{hot}}^{*}-T_{\mathrm{cold}}^{*}\right)\right\}$$

where *T*_hot_^*^, and *T*_cold_^*^ are the temperatures that maximize the rectangle below the Δ*S*_M_(*T*) curve.

*TEC* is calculated as

5 $$\mathrm{TEC}\left({\Delta T}_{\mathrm{lift}},\mu_{0}\Delta H\right)=\frac{1}{\Delta T_{\mathrm{lift}}}\max_{T_{\mathrm{mid}}\;}\left\{\int_{T_{\mathrm{mid}}-\Delta T_{\mathrm{lift}}/2}^{T_{\mathrm{mid}}+\Delta T_{\mathrm{lift}}/2}\Delta S_{M}\left(T,\mu_{0}\Delta H\right)dT\right\}$$

where *T*_mid_ is selected to maximize the integral.^56^

The values of the maximum of |Δ*S*_M_|^max^ and the refrigerant capacities *RC*-1, *RC*-2, and *RC*-3 at μ_0_Δ*H* = 2 and 5 T are nearly identical in both samples. However, the refrigerant capacities are slightly higher for ribbons, mainly due to the enhancement of the FWHM of the entropy change. The values for |Δ*S*_M_|^max^ for both bulk and ribbon samples are comparable to those reported for other hexagonal R_2_Fe_17_ materials, such as Er_2_Fe_17_, which is expected owing to the relatively similar saturation magnetization. Finally, Lu_2_Fe_17_, in both bulk and ribbon forms, exhibits larger refrigerant capacities due to the broad Δ*S*_*M*_(*T*) profile driven by the double magnetic transition.^10^

## Summary and Conclusions

We have investigated the effect of rapid solidification on the crystalline structure and the magnetic properties of the intermetallic Lu_2_Fe_17_ compound. Single-phase Lu_2_Fe_17_ ribbons, homogeneous in composition, with a disordered Th_2_Ni_17_-type crystalline structure, likewise that of the parent bulk alloy, were successfully produced. At low temperatures, the Lu_2_Fe_17_ ribbons exhibit a fan magnetic structure, alongside a helimagnetic phase, in a broader temperature range from 252 to 276 K, compared to 257 to 273 K for the bulk sample. The disorder introduced during the fabrication process causes the difference in the observed magnetic phase transition temperatures by rearranging Fe atoms and altering Fe–Fe distances. This results in shorter Fe–Fe separations, some of which fall below the critical exchange coupling length, thereby promoting antiparallel coupling between the magnetic moments of Fe atoms. Furthermore, it is important to note that our study reveals nearly zero thermal expansion over a broad temperature range below the transition to the incommensurate magnetic phase along the basal plane. Remarkably, we also observe negative thermal expansion along the *c*-axis of the hexagonal crystal structure, a rare and intriguing phenomenon that further highlights the strong coupling between magnetic ordering and lattice dynamics in Lu_2_Fe_17_ ribbons. The low-magnetic-field magnetocaloric effect emerges as a powerful tool for probing the two magnetic-field-dependent transitions in Lu_2_Fe_17_, which show up as two distinct peaks in the isothermal magnetic entropy change curves. Additionally, it provides valuable insights into the distribution of transition temperatures and strain effects. In this context, the broader profile of the Δ*S*_M_(*T*) curves for the melt-spun ribbons, compared to the bulk sample, directly reflects the broader distribution of ordering temperatures and the strain introduced by rapid solidification. Ultimately, we hope that the current investigation will assist the scientific community in demonstrating how low-magnetic-field isothermal magnetic entropy variations can reveal subtle yet significant changes related to the existence of different magnetic phase transitions in magnetocaloric materials or more complex magnetic systems.

## Supplementary Material



## Notes

Isothermal magnetization curves measured at *T* = 3 K (Figure S1); Isothermal magnetization curves along with the corresponding Arrott plots measured in the range 220–382 K and 210–269 K for both bulk and ribbon samples, respectively (Figure S2). These data complement the results presented in the manuscript file (PDF)
