# Supplementary material for: Delving into the Correlation between Magnetic and Lattice Degrees of Freedom from Magnetocaloric and Magnetovolume Effects in Lu2Fe17 Ribbons
Source: J Phys Chem C Nanomater Interfaces. 2025 Oct 7;129(41):18685–94. doi: 10.1021/acs.jpcc.5c04207 (PMC12536504; doi:10.1021/acs.jpcc.5c04207)
Supplement: Supplementary file 1 [file jp5c04207_si_001.pdf]

SUPPLEMENTARY INFORMATION

Delving Into the Correlation Between Magnetic and Lattice Degrees of Freedom from Magnetocaloric and Magnetovolume Effects in Lu_2Fe_{17} Ribbons

J.L. Garrido Álvarez[1], M.L. Arreguín-Hernández[2], C. Echevarría-Bonet[1], Pedro Gorria[1,3], I. Puente-Orench[4], F. Fauth[5], Jesús A. Blanco[1], J.L. Sánchez Llamazares[6**], Pablo Álvarez-Alonso[1,3]*

[1]Departamento de Física, Universidad de Oviedo, 33007, Oviedo, Spain.

[2]Centro de Nanociencias y Nanotecnología, Universidad Nacional Autónoma de México, AP 14, Ensenada 22860, Baja California, Mexico.

[3]IUTA, Universidad de Oviedo, 33203 Gijón, Spain.

[4]Institut Laue-Langevin, 71 Ave des Martyrs, CS 20156, 38042 Grenoble cedex 9, France.

[5]ALBA Synchrotron Light Source, 08290 Cerdanyola del Vallès, Barcelona, Spain.

[6]Instituto Potosino de Investigación Científica y Tecnológica A.C., San Luis Potosí S.L.P. 78216, Mexico.

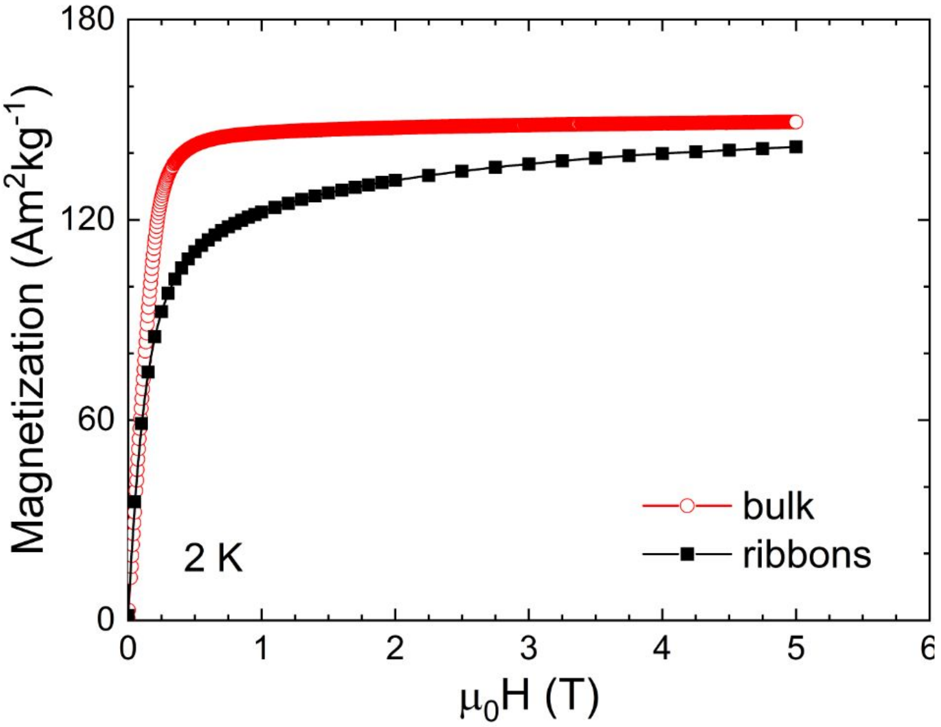

Fig. S1. Isothermal magnetization curves, $M(\mu_0 H)$, at 2 K up to a maximum magnetic field of 5 T. Lines are guides for the eyes.

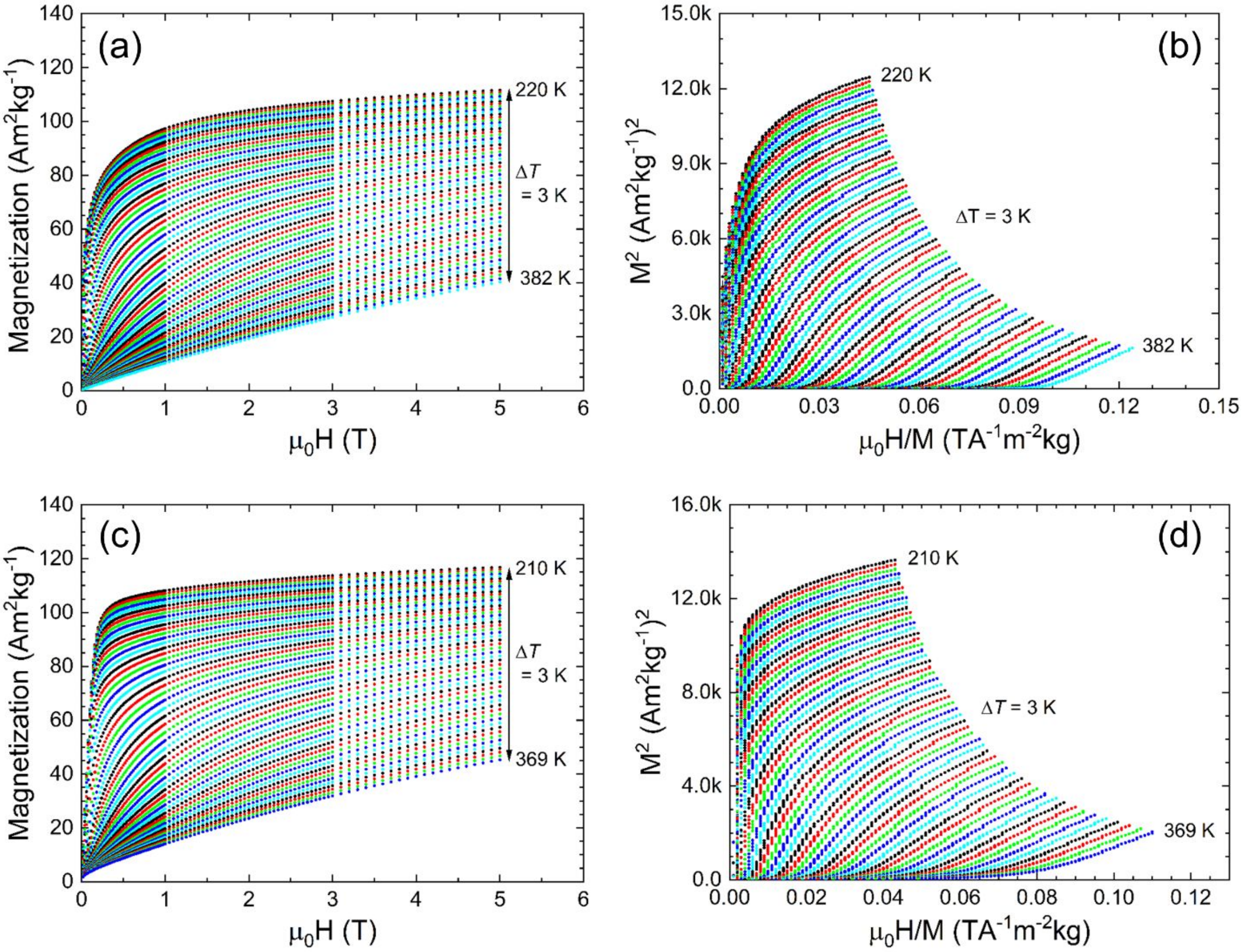

Fig. S2. Set of isothermal magnetization curves, $M(\mu_0 H)$, measured up to a maximum magnetic field of 5 T, along with the corresponding Arrott's plots for Lu_2Fe_{17} ribbons [(a) and (b)] and bulk alloy [(c) and (d)].
